# miR-2478 inhibits TGFβ1 expression by targeting the transcriptional activation region downstream of the TGFβ1 promoter in dairy goats

**DOI:** 10.1038/srep42627

**Published:** 2017-02-15

**Authors:** Zhuanjian Li, Xianyong Lan, Ruili Han, Jing Wang, Yongzhen Huang, Jiajie Sun, Wenjiao Guo, Hong Chen

**Affiliations:** 1 College of Animal Science and Veterinary Medicine, Henan Agricultural University, Zhengzhou, 450002, China; 2 College of Animal Science and Technology, Northwest A&F University, Shaanxi Key Laboratory of Molecular Biology for Agriculture, Yangling, Shaanxi 712100, P. R. China

## Abstract

In a previous study, miR-2478 was demonstrated to be up-regulated in dairy goat mammary glands during peak lactation compared with the dry period. However, the detailed mechanisms by which miR-2478 regulates physiological lactation and mammary gland development in dairy goats remain unclear. In this study, we used bioinformatics analysis and homologous cloning to predict the target genes of miR-2478 and selected *INSR, FBXO11, TGFβ1* and *ING4* as candidate target genes of miR-2478. Subsequently, by targeting the 5′UTR of the *TGFβ1* gene, we verified that miR-2478 significantly inhibited *TGFβ1* transcription and the Pearson’s correlation coefficient between miR-2478 expression and *TGFβ1* expression was −0.98. Furthermore, we identified the potential promoter and transcription factor binding regions of *TGFβ1* and analyzed the potential mechanisms of interaction between miR-2478 and *TGFβ1*. Dual-luciferase reporter assays revealed that two regions, spanning from −904 to −690 bp and from −79 to +197 bp, were transcription factor binding regions of *TGFβ1*. Interesting, the miR-2478 binding sequence was determined to span from +123 to +142 bp in the *TGFβ1* gene promoter. Thus, our results have demonstrated that miR-2478 binds to the core region of the *TGFβ1* promoter and that it affects goat mammary gland development by inhibiting *TGFβ1* transcription.

microRNAs (miRNAs) are 18–25-nucleotide-long, endogenous, single-stranded, non-coding RNA molecules that base-pair with target mRNAs in order to post-transcriptionally modulate gene expression through actions such as, translational repression or mRNA destabilization^1^,^2^. miRNAs are important mediators of numerous biological processes, including cell proliferation and apoptosis^3^, hormone secretion^4^, and tumor formation^5^.

A number of studies investigating miRNAs in farm animals have shown that several miRNAs play important roles in muscle development^6^, fat deposition^7^, oocyte maturation^8^ and early embryonic development^9^. However, functional studies of miRNAs in mammary tissues have only recently emerged. Indeed, in 2004, Liu *et al*.^10^ used microarrays to identify 23 novel, specific miRNAs in human mammary tissues, highlighting the need for studies of the mechanisms by which miRNAs regulate mammary gland development and lactation. Subsequently, numerous studies on the regulation of mammary gland development by miRNAs emerged, particularly studies focusing on the use of miRNAs as molecular markers of breast carcinogenesis. However, few studies have examined the normal regulatory functions of miRNAs in mammary glands, particularly in those of farm animals. In particular, human miR-125b has been reported to be expressed at high levels during all stages of mammary gland development but to be expressed at a low level in breast carcinoma tissue, suggesting that suppression of this miRNA negatively affects the differentiation of mammary gland epithelial cells^11^. Moreover, mouse miR-126–3p inhibits the proliferation of mammary gland epithelial cells and the expression of casein by binding to a target site in the 3′ untranslated region (UTR) of progesterone receptor (PGR)^12^. Further, in one study of ruminants, 59 miRNAs were isolated from bovine adipose and mammary gland tissues, among which miR-21, miR-23a, miR-24 and miR-143 were highly expressed in bovine mammary gland tissues^7^, and suppression of endogenous miR-24 expression reduced the rate of high-temperature-induced apoptosis of bovine mammary gland epithelial cell. In addition, some researchers have explored the molecular mechanisms of miRNAs in the regulation of lactation physiology and mammary gland development in the dairy goat^13^,^14^. In a previous study, we conducted comparative transcriptome profiling of miRNAs in dairy goat mammary gland tissues during both the dry period and peak lactation and identified 8 miRNAs, including miR-2478 (fold-change >1 and P-value < 0.01), that were up-regulated in the mammary gland during peak lactation compared with the dry period^14^. However, the roles and detailed mechanisms of miR-2478 in regulating physiological lactation and mammary gland development in dairy goats remain unclear.

In the present study, we predicted and screened four genes (insulin receptor, *INSR*; F-box protein 11, *FBXO11*; transforming growth factor beta 1, *TGFβ1*; and inhibitor of growth family member 4, *ING4*) as candidate targets of dairy goat miR-2478. Subsequently, we verified *TGFβ1* as a target gene of miR-2478 and inferred that this miRNA might participate in the negative regulation of dairy goat mammary gland development by targeting the 5′UTR of the *TGFβ1* gene. In addition, we identified the potential promoter and transcription factor binding regions of *TGFβ1* and examined the potential mechanisms of the miR-2478/*TGFβ1* interaction. The present study provides evidence that miR-2478 binds to the region of transcriptional activity downstream of the *TGFβ1* promoter and that it might synergistically regulate the inhibition of *TGFβ1* via the transcription factor recombination signal binding protein for immunoglobulin kappa J region (RBPJ) to affect goat mammary gland development.

## Materials and Methods

### Protocol approval

The experiments and animal care were performed according to the Regulations for the Administration of Affairs Concerning Experimental Animals (Ministry of Science and Technology, China, 2004) and were approved by the Institutional Animal Care and Use Committee (College of Animal Science and Technology, Northwest A&F University, China). All of the experiments and methods were carried out in “accordance” with the approved guidelines. The goats were allowed access to feed and water *ad libitum* under normal conditions, and all efforts were made to minimize their suffering.

### Identification of goat miR-2478 target genes

#### miRNA target prediction

miRNA target genes were predicted using TargetScan (http://www.targetscan.org/)^15^. The parameters were set according to the methods of Allen *et al*.^16^ and Schwab *et al*.^17^. The following criteria were applied for target gene prediction: (1) no more than four mismatches between the miRNA and target; (2) no more than two adjacent mismatches in the miRNA/target duplex; (3) no more than 2.5 mismatches at positions 1–12 of the miRNA/target duplex (5′ of the miRNA); (4) no mismatches at positions 10–11 of the miRNA/target duplex; (5) no adjacent mismatches at positions 2–12 of the miRNA/target duplex; and (6) minimum free energy of the miRNA/target duplex of more than 75% of that of the miRNA bound to its perfect complement. No data regarding mRNA:miRNA interactions in goats or sheep are available in the current version of TargetScan software. Therefore, the potential target genes of miR-2478 were predicted by referring to bovine mRNA:miRNA interactions and were screened according to the sequence characteristics of miR-2478 and the associated Gene Ontology (GO) terms^18^.

#### Cloning and verification of the corresponding target gene sequences

Seven primers for target genes were designed based on the sequences of bovine steroid receptor RNA activator 1 (*SRA1*, GenBank: AC_000164.1), *FBXO11* (GenBank: AC_000168.1), nuclear receptor corepressor 1 (*NCOR1*, GenBank: AC_000176.1), *TGFβ1* (GenBank: AC_000175), *ING4* (GenBank: AC_000162.1), inhibitor of growth family member 2, (*ING2*, GenBank: AC_000184.1) and *INSR* (GenBank: AC_000164.1). The primers, annealing temperatures, and fragment sizes are provided in Table S1. The corresponding target sequences were amplified from caprine genomic DNA. PCR amplification was performed in a 25 μL reaction mixture with the following cycling parameters:. 5 min at 95 °C, followed by 35 cycles of denaturation at 94 °C for 30 s, annealing for 30 s, and extension at 72 °C for 40 s, and a final extension step at 72 °C for 10 min. The amplification products were sub-cloned into a pMD18-T vector (Takara, Dalian, China), and then sequencing was performed. The caprine sequence identities of the miR-2478 binding sites were analyzed using BioXM 2.6software (Nanjing Agricultural University, Nanjing, China).

#### Plasmids

The potential target genes were further screened based on the results of sequence identity analysis of the miR-2478 binding sites. To generate wild-type and mutant vectors containing the miR-2478 binding sites of the candidate target genes (*FBXO11, TGFβ1, INSR* and *ING4*), primers were designed based on the results of candidate target gene sequencing in goat (Table 1). miR-2478 binding site mutants were generated via mutation of the target sequences of the miR-2478 seed region in the candidate target genes by overlap PCR. The PCR products for the wild-type and mutant target genes were cloned into multiple cloning regions of the vector psiCHECK-2 (Promega, Madison, WI, USA) using the *Not*I and *Xho*I restriction sites. The constructed vectors were sequenced to verify the base sequences of the miR-2478 binding and mutant sites.

#### HEK293T cell culture and co-transfection

HEK293T cells were cultured in Dulbecco’s modified Eagle’s medium (DMEM) supplemented with 10% fetal bovine serum (FBS) and 100 U/mL penicillin/streptomycin. The cells were then seeded into 24-well plates. Next, a miR-2478 mimic (2.0 μL) exhibiting miR-2478 activity was used to examine the effects of miRNA over-expression. The mimic was co-transfected into HEK293T cells with wild-type plasmids (psiCHECK-2-INSR, psiCHECK-2-FBXO11, psiCHECK-2-TGFβ1 and psiCHECK-2-ING4) or mutant plasmids (psiCHECK-2-INSR-mut, psiCHECK-2-FBXO11-mut, psiCHECK-2-TGFβ1-mut and psiCHECK-2-ING4-mut) (0.25 ng) according to the manufacturer’s instructions. An empty psiCHECK-2 plasmid was used as a negative control. Each sample was transfected in triplicate. At 48 h after transfection, cells were collected for use in subsequent dual-luciferase reporter assays.

#### Dual-luciferase reporter assay

Cells were lysed in passive lysis buffer (Promega, USA), and the lysates were assayed for reporter gene activity using a dual-luciferase assay system (Promega, USA) according to the manufacturer’s instructions. Luciferase signal was detected with a TD-20/20 luminometer (Turner Biosystems, Sunnyvale, CA, USA). Renilla luciferase signal was normalized to firefly luciferase signal, and normalized Renilla luciferase activity was compared between samples using the independent sample t-test.

#### Transfection with miR-2478 mimic or inhibitor and quantitative PCR (qPCR)

Dairy goat primary mammary gland epithelial cells (provided by Professor Jun Luo) were cultured in DMEM/F-12 medium supplemented with 10% FBS, 5 μg/mL bovine insulin, 10 ng/mL epidermal growth factor, 100 U/mL penicillin/streptomycin and 5 μg/mL hydrocortisone. The cells were grown at 37 °C and 5% CO_2_ and were transfected with 25 nM of a miR-2478 mimic or inhibitor or a negative control (NC) sequence (Qiagen, Hilden, Germany) using HiPerFect HTS Reagent according to the manufacturer’s instructions. Sterile ultra-pure water was employed as a negative control. Each sample was transfected in triplicate. Cells were directly collected after 24, 48 and 72 h, and total RNA was isolated from the cells using RNAiso Plus reagent (TaKaRa, Dalian, China). The RNA samples were subsequently reverse transcribed into cDNA using a PrimeScript^TM^ RT Reagent Kit with gDNA Eraser (TaKaRa, Dalian, China), according to the manufacturer’s instructions. Real-time PCR was performed using a standard SYBR Green PCR Kit (Takara, Dalian, China) and a BioRad CFX96 Real-Time PCR Detection System. Caprine miR-2478 expression patterns were verified via stem-loop RT-PCR according to Li *et al*.^14^. The miR-2478 mimic sequences were 5′-GUAUCCCACUUCUGACACCA-3′ (sense) and 5′-UGGUGUCAGAAGUGGGAUACUU-3′ (antisense), and the miR-2478 inhibitor sequence was 5′-UGGUGUCAGAAGUGGGAUAC-3′. In addition, the relative *TGFβ1* levels were determined using the following primers: caprine GAPDH: F: 5′-GCAAGTTCCACGGCACAG-3′ and R: 5′-GGTTCACGCCCATCACAA-3′; and caprine TGFβ1: F: 5′-GTGCTAATGGTGGAATACGG-3′ and R: 5′-CGCCAGGAATTGTTGCTATA-3′. 18S-snRNA and GAPDH were used as endogenous controls. Relative gene expression levels were determined using the 2^−ΔΔCt^ method.

### Analysis of the mechanism of *TGFβ1* regulation by miR-2478

#### Cloning of the caprine TGFβ1 gene and construction of 5′UTR sequence deletions

In a previous experiment, we showed that miR-2478 targeted the 5′UTR of *TGFβ1*. Therefore, to explore the mechanisms underlying the miR-2478/*TGFβ1* interaction, we cloned and sequenced fragments containing the entire coding region and partial intron of the caprine *TGFβ1* gene, as well as parts of the 5′ and 3′ flanking sequences. In addition, the promoter sequence of *TGFβ1* and the regulatory effects of miR-2478 binding to the 5′UTR were determined. Based on the bovine *TGFβ1* sequence (GenBank: NC_007316.5), seven pairs of primers for amplifying different fragments of the caprine *TGFβ1* gene were designed. The primers, annealing temperatures, and fragment sizes are shown in Table S1.

To verify the basic core promoter of the caprine *TGFβ1* gene, specific primers were designed based on the acquired caprine *TGFβ1* sequence to construct pGL3 vectors harboring deletions of varying lengths in the 5′UTR (Table S2). The PCR products were cloned into multiple cloning sites of a pGL3-Basic luciferase vector (Promega, WI, USA) using the *Kpn*I and *Hin*dIII restriction sites. Then, the constructed vectors were sequenced to verify the deleted sequences.

#### Cell culturing and transfection

The culture conditions for HEK293T cells were the same as described above. Prior to transfection, pGL3-Basic recombinant plasmids (500 ng) carrying sequences with deletions of different lengths were diluted with serum-free DMEM without antibiotics and mixed with pRL-CMV endogenous control plasmids (10 ng). Subsequently, the plasmid mixtures were transfected into HEK293T cells using Lipofectamine 2000.

Six groups were included in the experiment: a negative control group (pGL3-Basic and pRL-CMV), treatment group 1 (pGL3-TGFβ1 A and pRL-CMV), treatment group 2 (pGL3-TGFβ1B and pRL-CMV), treatment group 3 (pGL3-TGFβ1 C and pRL-CMV), treatment group 4 (pGL3-TGFβ1D and pRL-CMV) and treatment group 5 (pGL3-TGFβ1E and pRL-CMV). Each sample was transfected in triplicate. At 48 h after transfection, cells were collected for use in subsequent dual-luciferase reporter assays.

#### Bioinformatics analysis of the 5′UTR of TGFβ1

Potential *cis* elements in the *TGFβ1* 5′UTR were predicted using the following four online software platforms for the prediction of transcription factors and promoters: PROSCAN (http://www-bimas.cit.nih.gov/molbio/proscan/), Promoter 2.0 (http://www.cbs.dtu.dk/services/Promoter), TFSEARCH (http://mbs.cbrc.jp/research/db/TFSEARCH.html) and MatInspector (http://www.genomatix.de/matinspector.html).

### Statistical analysis

Statistical analyses of normalized Renilla luciferase activity and the relative expression levels of miR-2478 and *TGFβ1* were performed using SPSS version 20.0. The correlation between miR-2478 expression and *TGFβ1* mRNA expression was assessed using Pearson’s correlation analysis. In addition, the sample data were compared using the independent sample t-test and one-way ANOVA, followed by Dunnett’s test, in GraphPad Prism 5 (GraphPad Software, San Diego, CA, USA). The data are expressed as the mean ± SE.

## Results

### Prediction of miR-2478 target genes and experimental verification of miR-2478 binding sites in these genes in goat

To further elucidate the role of miR-2478 in regulating mammary gland development in dairy goats, we used TargetScan^15^ (http://www.targetscan.org/) to predict the target genes regulated by miR-2478. Based on their high scores, sequence characteristics and GO terms, we selected seven potential targets: *SRA1, FBXO11, NCOR1, TGFβ1, ING2, ING4* and *INSR. TGFβ1* is associated with the GO terms “transforming growth factor beta receptor signaling pathway” and “secretion by cell,” and *ING2* is associated with the terms “transforming growth factor beta receptor signaling pathway,” “regulation of biosynthetic process” and “biological regulation.” Notably, the *TGFβ* receptor signaling pathway plays important roles in mammary gland development, differentiation and degeneration. In addition, *NCOR1* is associated with the GO terms “lipid metabolic process,” “lipid transport,” “lipid localization” and “fatty acid transport,” which play important roles during lactation.

**Figure 1:**
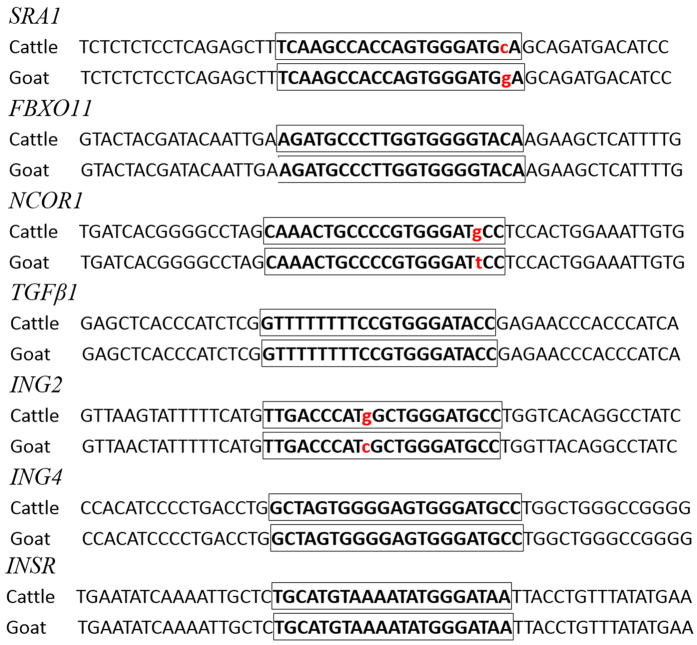
Target sequence alignment of miR-2478 sequences between cattle and goat. The miR-2478 target binding sequences are indicated by the boxes. Mismatched bases are indicated by red lowercase letters.

As the prediction of target genes was based on bovine genomic sequences, we suspected that the corresponding sequences in the caprine genome might exhibit some differences. Thus, the corresponding sequences of the miR-2478 binding sites in the potential target genes were amplified from caprine genomic DNA and sequenced by homologous cloning. Nucleotide sequence alignment analysis indicated base mismatches in the miR-2478 binding sites in goat *SRA1, NCOR1* and *ING2* compared with the corresponding sequences in the bovine genes, while the miR-2478 binding sites in the other four potential target genes were the same in goat and cattle (Fig. 1). Therefore, the remaining four genes (*INSR, FBXO11, TGFβ1* and *ING4*) were screened as candidate targets of dairy goat miR-2478. The miR-2478 binding sites of three of the four candidate targets were located in the 3′UTR, whereas that of *TGFβ1* was located in the 5′UTR.

### miR-2478 binds to the 5′UTR of the *TGFβ1* gene

**Figure 2:**
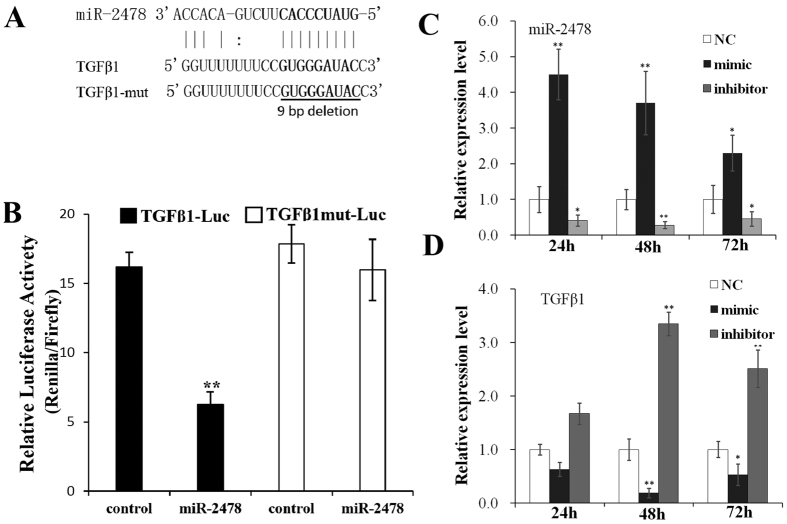
miR-2478 inhibits *TGFβ1* transcription by binding to its 5′UTR. (**A**) Predicted miR-2478 target sequence in goat *TGFβ1*. The seed region (in bold) was deleted in the psiCHECK-2-TGFβ1-mut vector. (**B**) Dual-luciferase reporter assay verified that *TGFβ1* is a target gene of miR-2478. A recombinant psiCHECK-2 vector or psi-CHECK2 vector was co-transfected into HEK293T cells with a miR-2478 mimic or a negative control. After 48 h, the cells were lysed, and luciferase assays were performed. (**C**) qPCR confirmed the effective transfection of the miR-2478 mimic or inhibitor into mammary gland epithelial cells. Sterile ultra-pure water was used as a negative control. Cells were harvested at 24, 48 and 72 h after transfection. (**D**) qPCR was performed to detect the *TGFβ1* mRNA levels in mammary gland epithelial cells after transfection with the miR-2478 mimic, inhibitor or NC. The data are presented as the mean ± SE (n = 3) (*P < 0.05; **P < 0.01).

**Figure 3:**
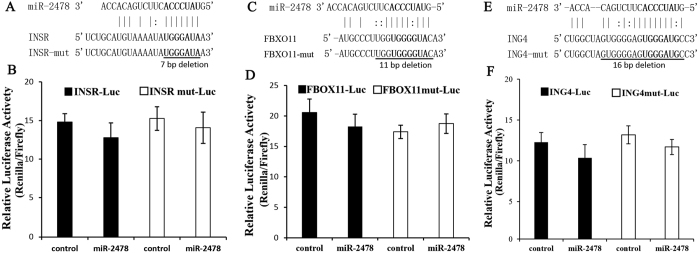
(**A**,**C** and **E**) Predicted miR-2478 target sequences in goat INSR, FBXO11 and ING4, respectively. The seed region (in bold) was deleted in the mutant recombinant psiCHECK-2 vector. **(B**,**D** and **F**) Dual-luciferase reporter assays showing that INSR, FBXO11 and ING4, respectively, are not target genes of miR-2478. A recombinant psiCHECK-2 vector or psi-CHECK2 vector was co-transfected with a miR-2478 mimic or negative control into HEK293T cells. After 48 h, the cells were lysed, and luciferase assays were performed. The data are presented as the mean ± SE (n = 3).

A dual-luciferase reporter system was used to identify the target genes of miR-2478. The luciferase activity of HEK293T cells co-transfected with the miR-2478 mimic and psiCHECK-2-TGFβ1 5′UTR vector was significantly decreased compared with that of cells transfected only with the negative control psiCHECK-2-TGFβ1 5′UTR vector (*P* < 0.01), while luciferase activity was recovered in cells transfected with the psiCHECK-2-TGFβ1-mut vector (*P* < 0.01) (Fig. 2A and B), indicating that miR-2478 directly targets *TGFβ1* by binding to its 5′UTR. *INSR, FBXO11* and *ING4* were not found to be positive target genes, as no significant differences in luciferase activity were observed between cells co-transfected with the mimic and psiCHECK-2-INSR, psiCHECK-2-FBXO11 or psiCHECK-2-ING4 and negative control cells (*P* > 0.05) (Fig. 3). A previous study has reported that miR-2478 expression is up-regulated in dairy goat mammary glands during peak lactation, whereas *TGFβ1* gene expression is down-regulated^14^. These results demonstrate that miR-2478 binds to the 5′UTR of the *TGFβ1* gene (from +123 bp to +142 bp) (Fig. 2A).

### miR-2478 significantly inhibits *TGFβ1* transcription

The dual-luciferase reporter assay confirmed the interaction between miR-2478 and the 5′UTR of the *TGFβ1* gene. Thus, we inferred that miR-2478 might affect the transcription of *TGFβ1*. To verify this hypothesis, we examined changes in *TGFβ1* mRNA expression in dairy goat primary mammary gland epithelial cells after transfection of a miR-2478 mimic or inhibitor for 24, 48 or 72 h. qPCR assays showed that the expression of mature miR-2478 was significantly higher at 24 and 72 h after transfection of the miR-2478 mimic than that in control cells (*P* < 0.01), indicating that the miR-2478 mimic was successfully transfected into the mammary gland epithelial cells (Fig. 2C). In addition, the expression of endogenous mature miR-2478 was significantly lower at 24, 48 and 72 h after transfection of the miR-2478 inhibitor than that in the control (*P* < 0.01 or *P* < 0.05), indicating that the miR-2478 inhibitor suppressed the expression of endogenous miR-2478 in mammary epithelial cells (Fig. 2C). qPCR after 48 h of over-expression of miR-2478 in dairy goat mammary gland epithelial cells revealed that the *TGFβ1* mRNA level was significantly down-regulated compared with that in the control cells (*P* < 0.01) (Fig. 2D). In addition, the level of *TGFβ1* mRNA was significantly up-regulated at 48 and 72 h after transfection of the miR-2478 inhibitor (*P* < 0.01) (Fig. 2D). These findings demonstrate that miR-2478 significantly inhibits *TGFβ1* transcription by binding to the 5′UTR of the *TGFβ1* gene (from +123 bp to +142 bp).

### Expression analysis and investigation of the conservation of the *TGFβ1* gene in goat

**Figure 4:**
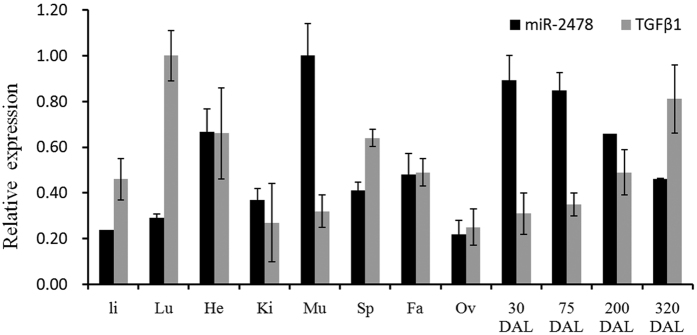
Profiling analysis of the TGFβ1 gene and miR-2478 in different tissues.

In a previous study, we investigated the expression patterns of miR-2478 in the skeletal muscle (Mu), heart (He), liver (Li), kidney (Ki), spleen (Sp), lung (Lu), inner fat (Fa), ovary (Ov) and mammary gland at four developmental stages (at 30 days after lambing (DAL), 75 DAL, 200 DAL, and 320 DAL)^14^. We also performed profiling analysis of miR-2478 targeting of the *TGFβ1* gene in these tissues. *TGFβ1* gene expression was higher at 30 and 75 DAL (peak lactation) than at 320 DAL (dry period) (Fig. 4), revealing that this gene is related to mammary gland development. Furthermore, the correlation between miR-2478 expression and *TGFβ1* mRNA expression was assessed by Pearson’s correlation analysis using SPSS yielding a correlation coefficient of −0.98 (*P* = 0.017). These findings demonstrate that the expression levels of miR-2478 and *TGFβ1* mRNA in the goat mammary gland are inversely correlated during lactation, implying that miR-2478 may be a major regulator of *TGFβ1* mRNA expression during lactation.

**Figure 5:**
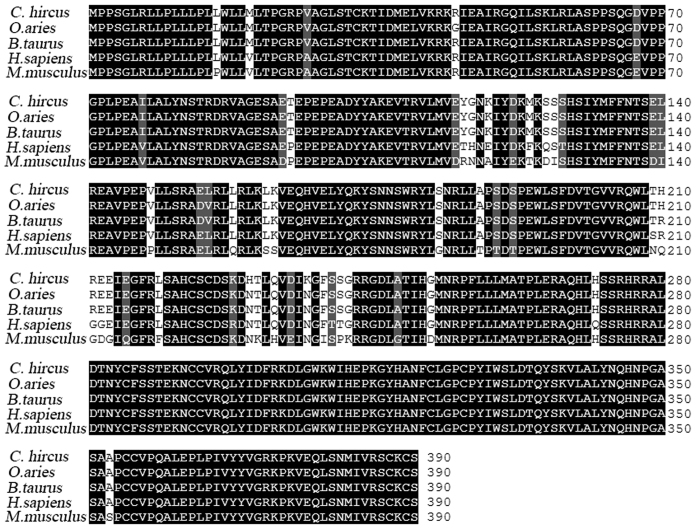
Comparison of sequence homology among goat, sheep, cattle, human, and mouse TGFβ1 amino acid sequences. The amino acid sequences were derived from the following reference sequences: NP_001009400.1 (sheep), NP_001159540.1 (cattle), NP_000651.3 (human), and NP_035707.1 (mouse).

The caprine *TGFβ1* gene (length of 1971 bp) was cloned and sequenced, with the length of 1971 bp. Alignment of the caprine *TGFβ1* cDNA with the corresponding coding sequences (CDSs) from other mammalian species showed similarities of 98.04% with sheep, 98.30% with cattle, 89.86% with humans and 83.38% with mice. At the protein level, the caprine amino acid sequence shared 99.12, 99.12, 97.83 and 95.37% identity with sheep, cattle, humans and mice, respectively, as shown in Fig. 5. These results indicate that the *TGFβ1* gene is highly conserved, implying that the function of this gene is consistent among mammals.

### The mechanism of miR-2478 in inhibiting *TGFβ1* transcription

**Figure 6:**
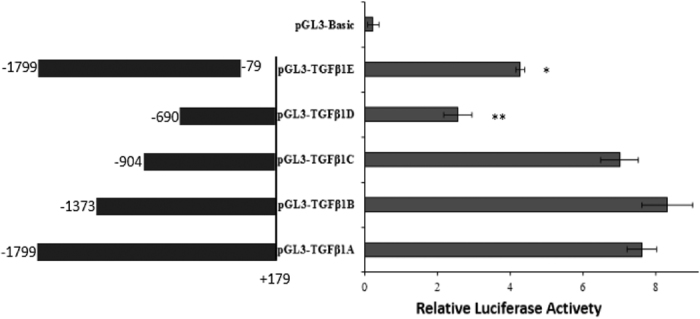
Luciferase activity assays for a series of truncated sequences of the TGFβ1 5′UTR in HEK293T cells. pGL3-TGFβ1A/B/C/D/E or a pGL3-Basic vector was co-transfected with a pRL-CMV vector into HEK293T cells. Then, the cells were lysed, and luciferase assays were performed after 48 h. The pGL3-Basic vector was used as a negative control. The data are presented as the mean ± SE (n = 3). (*P < 0.05; **P < 0.01).

**Figure 7:**
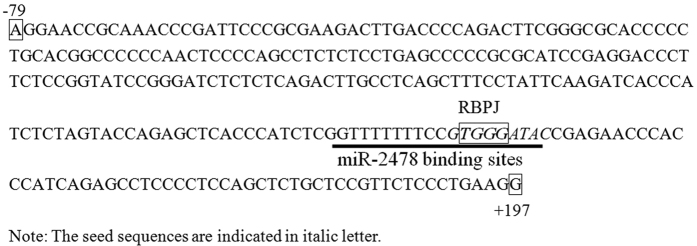
The core region of the TGFβ1 gene promoter and miR-2478 binding site region.

While the above results demonstrated that miR-2478 binds to the 5′UTR of *TGFβ1* to exert a regulatory function, the mechanisms underlying the miR-2478/*TGFβ1* 5′UTR interaction remained unclear. Thus, to examine this interaction, potential promoters and transcription factor binding regions in the 5′UTR of *TGFβ1* were assessed. Various deletion vectors for the goat TGFβ1 5′UTR were constructed and designated pGL3-TGFβ1 A, pGL3-TGFβ1B, pGL3-TGFβ1 C, pGL3-TGFβ1D and pGL3-TGFβ1E. The luciferase activities of HEK293T cells co-transfected with the pGL3-TGFβ1 A/B/C/D/E recombinant vectors or the endogenous control vector pRL-CMV were significantly higher than that of cells transfected with the negative control vector (pGL3-Basic and pRL-CMV; *P* < 0.05 or *P* < 0.01), indicating that the corresponding sequences all initiated reporter gene transcription, but to different extents (Fig. 6). Among the five recombinant vectors, pGL3-TGFβ1B resulted in the highest luciferase activity (8.32 ± 0.71), and pGL3-TGFβ1D resulted in the lowest activity (2.57 ± 0.39). Considering the luciferase activity of pGL3-TGFβ1 A as a reference (100%), the relative luciferase activities of the other four recombinant vectors (pGL3-TGFβ1B-E) were 109.04%, 91.87%, 33.68% and 56.09%, respectively (Table 2). Statistical analysis showed that the transcriptional activity of pGL3-TGFβ1D was significantly lower than the activities of pGL3-TGFβ1 A/B/C (*P* < 0.01) and pGL3-TGFβ1E (*P* < 0.05). In addition, the transcriptional activity of pGL3-TGFβ1E was significantly lower than those of pGL3-TGFβ1 A/B/C (*P* < 0.05) but was significantly higher than that of pGL3-TGFβ1D (*P* < 0.05). No significant differences in transcriptional activity were observed among pGL3-TGFβ1 A/B/C. The results of dual-luciferase reporter assays revealed that two regions spanning from −904 to −690 bp and from −79 to +197 bp were transcription factor binding regions in the *TGFβ1* 5′UTR. The above-mentioned results demonstrated that miR-2478 bound to the region from +123 to +142 bp in the *TGFβ1* 5′UTR (GGTTTTTTTCCGTGGGATAC) (Fig. 2A). It is interesting that this miR-2478 binding region (+123 to +142 bp) is located in the core region of the *TGFβ1* gene promoter (from −79 to +197 bp) (Fig. 7). Taken together, our results demonstrate that miR-2478 binds to the core region of the *TGFβ1* promoter and affects goat mammary gland development by inhibiting *TGFβ1* transcription.

### Transcription factor prediction for the *TGFβ1* 5′UTR and regulatory analysis of miR-2478

The *TGFβ1* promoter was predicted using three online software platforms: PROSCAN, Promoter 2.0 and MatInspector (Table S3). The overlapping predictions of PROSCAN and MatInspector showed a region of transcriptional activity downstream of the *TGFβ1* transcription start site, and those of PROSCAN and Promoter 2.0 showed a region of transcriptional activity approximately −1000 bp upstream of the transcription start site. These two predictions are approximately consistent with the previous experimental identification of transcription factor binding regions in the *TGFβ1* 5′UTR (two regions from −904 to −690 bp and from −79 to +197 bp). Next, we predicted the transcription factor binding sites in the two regions of the *TGFβ1* 5′UTR using TFSEARCH. A total of 21 binding sites were predicted, including 8 Sp1 sites, 3 GCF sites, 2 T-Ag sites, 1 PEBP2 site, 1 ETF site and 1 AP-2 site, spanning from −731 to −981 bp. The region from +29 to +279 bp was predicted to contain 9 Sp1 sites, 2 AP-2 sites, 1 GCF site, 1 NF-κB site and 1 T-Ag site. The prediction results indicated that these two regions are enriched in transcription factor binding sites. Moreover, a *cis*-acting element of the transcription factor RBPJ was identified in the core region from +135 to +138 bp (*TGGG*), while the binding seed region of miR-2478 was detected at +134 to +142 bp (G*TGGG*ATAC) (Fig. 7). Previous studies have shown that RBPJ is a critical transcription factor in mammals. Thus, these results indicate that the binding of miR-2478 to *TGFβ1* (seed sequence: G*TGGG*ATAC) may prevent the transcription factor RBPJ from binding to the promoter (*cis*-acting element: *TGGG*), thereby inhibiting *TGFβ1* transcription.

## Discussion

miRNAs have been identified in mice, humans, bovines and goats and in mammalian mammary glands at various developmental stages, suggesting that multiple miRNAs play important roles in the differential expression of genes during mammary development and lactation. miR-155 affects cell proliferation and apoptosis by targeting *RhoA*, and this process is mediated by TGFβ signaling pathways^19^. Jie^20^ has confirmed that the targeting of 3′UTR sequences in *GHR* by miR-139 in bovine mammary gland epithelial cells might play a role in the activity of ductal epithelial cells during lactation. However, the function and potential mechanism of miR-2478 in regulating mammary gland development in dairy goats have not previously been explored.

In the present study, we predicted the target genes of goat miR-2478 using the TargetScan program. Previous predictions of miRNA target genes have been focused on the 3′UTR. However, studies have recently shown that in addition to traditional 3′UTR targeting, a number of 5′UTR and amino acid CDS sites are also targeted in mammals^21^,^22^. Although high percentages of reads mapping to CDS sites (>25%) and 5′UTRs (>1%) have been reported, most reads match to the 3′UTR, confirming that miRNAs preferentially bind to targets in the 3′UTR but that 5′UTR/CDS-mediated interactions are also significant. Therefore, we considered the three potential miRNA target regions in the prediction of goat miR-2478 targets. We preliminarily selected seven potential targets and ultimately screened four of these genes (*INSR, FBXO11, TGFβ1* and *ING4*), which negatively regulate mammary gland development and physiological lactation, as candidate targets of dairy goat miR-2478 by PCR verification using goat genomic DNA and by sequence identity analysis of miR-2478 binding sites in goat and bovine (Fig. 1). miR-2478 binding sites were identified in the 5′UTR of *TGFβ1*, while in the other three target genes, traditional sites in the 3′UTR were detected. These results demonstrate that *TGFβ1* is a target gene of miR-2478 (Fig. 2A and B). Subsequently, we found that over-expression of miR-2478 in dairy goat mammary gland epithelial cells resulted in significant down-regulation of the *TGFβ1* mRNA level, while inhibition of endogenous miR-2478 expression resulted in significant up-regulation of *TGFβ1* mRNA (Fig. 2C and D), demonstrating that miR-2478 significantly inhibited *TGFβ1* transcription by binding to its 5′UTR. The potential regulation of mRNAs by miRNAs through binding sites in their 5′UTRs was demonstrated early on^23^. Many validated examples of naturally occurring 5′UTR miRNA targets exist in the literature to date^21^,^22^,^23^,^24^,^25^,^26^,^27^,^28^. Likewise, the present study has provided additional data in support of 5′UTR targeting by a miRNA, miR-2478, which resulted in a reduction in *TGFβ1* mRNA abundance.

We also investigated the conservation of the *TGFβ1* gene in mammals, including goat, sheep, cattle, humans and mice (Fig. 5). These results showed that the *TGFβ1* gene is highly conserved, implying that the function of this gene is consistent, among mammals. *TGFβ1* plays important roles in mammary gland development, differentiation and degeneration^29^. In microfabricated mammary gland tissue, branching has been shown to be inhibited at sites with high concentrations of TGFβ1^30^. WNT5A acts downstream of TGFβ1 *in vivo* and is required for TGFβ-mediated inhibition of mammary branching morphogenesis^31^,^32^,^33^. Increasing evidence indicates that TGFβ1 functions as a paracrine or autocrine epithelial cell inhibitory factor and as an apoptosis-inducing factor in mammary glands. In mice, TGFβ1 is a potent inhibitor of cell proliferation, and it has been shown to suppress alveolar development and lactogenesis^34^,^35^. Overe-xpression of *TGFβ1* has been reported to lead to inhibition of mammary gland development *in vivo*^36^. In addition, TGFβ1 has been demonstrated to be an antiproliferative and apoptogenic factor in dairy cattle mammary epithelial cells^37^. Thus, *TGFβ1* overexpression may decrease the autophagic apoptosis of mammary epithelial cells in cattle. TGFβ1 belongs to a group of intramammary autocrine/paracrine inhibitors of bovine mammary epithelial cell growth and inducers of apoptosis^38^. Moreover, TGFβ1 induces apoptosis of bovine mammary epithelial cells through the ALK-5-Smad2/3 pathway and promotes bovine mammary fibroblast proliferation through the ERK1/2 pathway to maintain normal development and lactation in the mammary glands^39^,^40^. Thus, TGFβ1 may act as a growth suppressor, exerting negative effects on mammary epithelial cell growth and also suppressing milk secretion^41^.

In addition, Wareski *et al*.^42^ investigated the expression of apoptosis-related proteins in the mammary glands of dairy goats during the lactation cycle (peak lactation, late lactation and drying off) and observed that the expression of the examined proteins was increased during late lactation and the dry period, suggesting that these proteins are involved in the induction, regulation and execution of programmed cell death during mammary gland involution. Based on these data, we inferred that miR-2478 might play an important role in mammary gland development in dairy goats by targeting the 5′UTR of the *TGFβ1* gene.

To further explore how miR-2478 regulates the transcription of *TGFβ1* by binding to its 5′UTR, we identified the potential core promoter and transcription factor binding regions of *TGFβ1* and analyzed the potential mechanisms of interaction between miR-2478 and *TGFβ1*. The experimental results indicated that two sequences spanning from −904 to −690 bp and from −79 to +197 bp might act as transcription factor binding regions in *TGFβ1* (Fig. 6), and this was also confirmed through promoter/transcription factor prediction for this gene (Table S3). It is interesting that the binding region of miR-2478 (+123 to +142 bp) is located in the core region of the *TGFβ1* gene promoter (−79 to +197 bp). In addition, we observed that the miR-2478 binding sequence was located in the region of transcriptional activity downstream of the *TGFβ1* promoter. Moreover, our predictions showed that the seed sequence of miR-2478 that binds to *TGFβ1* (TGGG) is a *cis*-acting element of the inhibitory transcription factor RBPJ. Thus, we speculate that miR-2478 inhibits *TGFβ1* expression at the transcriptional level by preventing the transcription factor RBPJ from binding to the promoter.

Accumulating evidence indicates that some miRNAs bind to the promoter regions of target genes to regulate their mRNA levels^43^,^44^,^45^,^46^,^47^. We and others have demonstrated that miRNAs complementary to gene promoters are potent silencers of target gene expression in mammalian cells at the transcriptional level. The miRNA-promoter interaction resource is a public database containing over 15 million predicted miRNA target sites located within human promoter sequences^48^. For example, the scanning of miRNA target sequences in transcription start sites using bioinformatic techniques has revealed that miR-320 acts at the *POLR3D* promoter and that it plays a *cis*-regulatory role in the silencing of gene expression at the transcriptional level^43^. In addition, Place has provided evidence of a promoter-targeting miRNA by demonstrating that the transfection of cells with miR-373 induces the expression of *CDH1* and *CSDC2,* which possess complementary promoter sequences^44^. Sequence-specific recognition of *PGR* gene promoters by miR-423 may occur simultaneously with binding by protein transcription factors^45^. Moreover, miR-130a functions as a suppressor that modulates promoter activity even in the presence of other direct or indirect transcriptional modulators^46^. These observations suggest that miRNA-promoter interactions may be a natural and general mechanism for regulating gene transcription. In our study, miR-2478 bound to the promoter region of its target gene, *TGFβ1*, and regulated its expression at the transcriptional level. Moreover, a *cis*-acting element of the transcription factor RBPJ was predicted to be located in the *TGFβ1* promoter region.

Knowledge of the mechanisms underlying the regulatory effects of miRNAs on target genes is increasing. Transcription factors and miRNAs have been frequently detected in numerous transcriptional regulatory network motifs. As cancer research expands, transcription factors, miRNAs and their equivalent target genes are emerging in an increasing number of studies^49^,^50^,^51^,^52^. Thus, we infer that the transcription factor RBPJ, miR-2478 and their shared target gene *TGFβ1* may interact to achieve transcriptional regulation. However, this hypothesis requires further verification.

In conclusion, we have revealed that miR-2478 significantly inhibits *TGFβ1* transcription by targeting the 5′UTR of the TGFβ1 gene. Furthermore, we have identified the potential promoter and transcription factor binding regions of *TGFβ1* and have analyzed the potential interaction mechanisms of miR-2478/*TGFβ1*. Previous studies have indicated that the transcription factor binding regions of *TGFβ1* include two regions spanning from −904 to −690 and from −79 bp to +197 bp. In addition, based on the prediction that the seed sequence of miR-2478 that binds to *TGFβ1* (TGGG) is a *cis*-acting element of the inhibitory transcription factor RBPJ, we suggest that the binding of miR-2478 to *TGFβ1* (seed sequence: G*TGGG*ATAC) may prevent the transcription factor RBPJ from binding to the promoter (*cis*-acting element: *TGGG*) and thereby inhibiting *TGFβ1* transcription.

## Additional Information

**How to cite this article**: Li, Z. *et al*. miR-2478 inhibits TGFβ1 expression by targeting the transcriptional activation region downstream of the TGFβ1 promoter in dairy goats. *Sci. Rep.* **7**, 42627; doi: 10.1038/srep42627 (2017).

**Publisher's note:** Springer Nature remains neutral with regard to jurisdictional claims in published maps and institutional affiliations.

## Supplementary Material

Supplementary Information

## Figures and Tables

**Table 1:** Primers used to construct wild-type and mutant vectors containing the miR-2478 binding sites in candidate target genes.

Gene	Primers (5′−3′)	Sizes (bp)
**Wild type**		
*INSR*	F1: ccgCTCGAGGCTTATGCGGAAATCAACTC	301
	R1: atttGCGGCCGCAACAAATACCCACAACACCC	
*FBXO11*	F2: ccgCTCGAGGCAAGATACAAAGGAAGAG	502
	R2: atttGCGGCCGCTTGGCATCAAGAATTATACAC	
*TGFβ1*	F3: ccgCTCGAGTCTCAGACTTGCCTCAGCTTTCC	443
	R3: atttGCGGCCGCATGCGCTTCCGCTTCACC	
*ING4*	F4: ccgCTCGAGCACGCTGCTCCCAAGAACGA	305
	R4: atttGCGGCCGCAAAGGACAGCGGGCAACACC	
**Mutation**		
*INSR-mut*	F1: ccgCTCGAGGCTTATGCGGAAATCAACTC	294
	OR: CCGAATTTTCATATAAACAGGTAATTATTTTACATGCAGAGC	
	OF: GCTCTGCATGTAAAATAATTACCTGTTTATATGAAAATTCGG	
	R1: atttGCGGCCGCAACAAATACCCACAACACCC	
*FBXO11-mut*	F2: ccgCTCGAGGCAAGATACAAAGGAAGAG	491
	OR: GTCCATCAAAATGAGCTTCTTAGGGCATCTTCAATTGTATCG	
	OF: CGATACAATTGAAGATGCCCTAAGAAGCTCATTTTGATGGAC	
	R2: atttGCGGCCGCTTGGCATCAAGAATTATACAC	
T*GFβ1-mut*	F3: ccgCTCGAGTCTCAGACTTGCCTCAGCTTTCC	434
	OR: TCTGATGGGTGGGTTCTCGGGAAAAAAACCGAGATGG	
	OF: CCATCTCGGTTTTTTTCCCGAGAACCCACCCATCAGA	
	R3: atttGCGGCCGCATGCGCTTCCGCTTCACC	
*ING4-mut*	F4: ccgCTCGAGCACGCTGCTCCCAAGAACGA	289
	OR: TGACCCCCGGCCCAGCCAGTAGCCAGGTCAGGGGATGT	
	OF: ACATCCCCTGACCTGGCTACTGGCTGGGCCGGGGGTCA	
	R4: atttGCGGCCGC AAAGGACAGCGGGCAACACC	

Note: The attached nucleotides are indicated in lowercase, and the restriction sites for *Not*I and *Xho*I are underlined.

**Table 2:** Relative luciferase activity assays for *TGFβ1* 5′UTR sequences in HEK293T cells.

Group	Relative luciferase activity	Normalized to pGL3-TGFβ1 A
pGL3-TGFβ1 A	7.63 ± 0.41^a^	100%
pGL3-TGFβ1B	8.32 ± 0.71^a^	109.04%
pGL3-TGFβ1 C	7.01 ± 0.51^a^	91.87%
pGL3-TGFβ1D	2.57 ± 0.39^bc^	33.68%
pGL3-TGFβ1E	4.28 ± 0.12^ad^	56.09%
pGL3-Basic	0.23 ± 0.16	3.01%

Note: compared with pGL3-Basic, a: *P* < 0.01, b: *P* < 0.05; compared with pGL3-TGFβ1 A, c: *P* < 0.01, d: *P* < 0.05.
