# Erratum: miR-2478 inhibits TGFβ1 expression by targeting the transcriptional activation region downstream of the TGFβ1 promoter in dairy goats

**DOI:** 10.1038/srep46966

**Published:** 2018-04-06

**Authors:** Zhuanjian Li, Xianyong Lan, Ruili Han, Jing Wang, Yongzhen Huang, Jiajie Sun, Wenjiao Guo, Hong Chen

Scientific Reports 7: Article number: 42627; 10.1038/srep42627 published online: 02 15 2017; updated: 04 06 2018.

The original PDF version of this Article contained an error in the order of corresponding authors. This has now been corrected in the PDF version of this Article; the HTML version was correct from the time of publication.

