# Supplementary material for: miR-2478 inhibits TGFβ1 expression by targeting the transcriptional activation region downstream of the TGFβ1 promoter in dairy goats
Source: Sci Rep. 2017 Feb 15;7:42627. doi: 10.1038/srep42627 (PMC5309801; doi:10.1038/srep42627)
Supplement: Supplementary Information [file srep42627-s1.pdf]

miR-2478 inhibits TGFβ1 expression by targeting the transcriptional activation region downstream of the TGFβ1 promoter in dairy goats

Zhuanjian Li[1, 2], Xianyong Lan [2], Ruili Han[1], Jing Wang[1], Yongzhen Huang[2], Jiajie Sun[2], Wenjiao Guo[2], Hong Chen[2*]

Table S1 Homologous cloning primers for the candidate target genes

Locus	Primer (5'-3')	Size (bp)	T_m °C
**Primers of candidate target genes**			
INSR	F1: GCTTATGCGGAAATCAACTC<br>R1: AACAAATACCCACAACACCC	280	57
SRA1	F2: GAACAATGGGCTGGAGGGAA<br>R2: CATGCCAAGGCACAGGAGAT	480	61
FBXO11	F3: GCAAGATACAAAGGAAGAG<br>R3: TTGGCATCAAGAATTATACAC	481	56
NCOR1	F4: ACAGTGCAGCAGTAGTTATCGG<br>R4: GCTTTGGGAGCTTCGTGGAGA	461	59
TGFβ1	F5: CCGACTCCCGCGAAGACTTGA<br>R5: GCGTCAGCATTAGCAGCCACA	476	56
ING2	F6: TAGTGCTTCATAAGGGAGTT<br>R6: AGACACCAAACAGCACCAAT	379	55
ING4	F7: CACGCTGCTCCCAAGAACGA<br>R7: AAAGGACAGCGGGCAACACC	284	53
**Primers of cloning *TGFβ1* gene**			
TGFβ1-5UTR	F8: CGGAGATGCCATCTACAG<br>R8: GGGTGATCTTGAATAGGAAAG	1661	56
TGFβ1-5UTR	F9: CTCCCTCCCTGCCGCCTATA<br>R9: GATGCGCTTCCGCTTCACC	620	53
TGFβ1-exon1	F10: CGCAAACAGACCCTCCTACC<br>R10: ATCCCGTCGCCCTCTAAAA	729	58
TGFβ1-exon2	F11: CCAACCCAGAGTTCTTCGA<br>R11: CTGTGCTCCCTCATCTTTCA	360	55
TGFβ1-exon3	F12: GGCAGCCTCTACCCACTATCT<br>R12: CCTATCCCTCTGCTTTGAACAC	631	59
TGFβ1-exon4,5	F13: TAGCCAAAGACAAATGTTAGAGC<br>R13: GACCTTCCAATACTGAAGTCCC	976	56
TGFβ1-exon6	F14: AGATAACGCTTATGAGGATG<br>R14: CCATCTTGCCAATTCATCT	502	60
TGFβ1-exon7	F15: AGTGAGATACAAGCCAAGAG<br>R15: CAGTAGTAGTGAGGAACATAAC	634	57

Table S2 Information on primers for fragments of the TGFβ1 5’UTR with deletions of different lengths

Fragment	Primer (5'-3')	Size (bp)	Location
A	F1: **GG***GGTACC*CTTCACCTTTCCACCCTCCCTC<br>R1: **CCC***AAGCTT*CCTTCAGGGAGAACGGAGCAGA	1996	-1799 ~ +197
B	F2: **GG***GGTACC*CATGGGCTTTGGAGACTGTGAGG<br>R2: **CCC***AAGCTT*CCTTCAGGGAGAACGGAGCAGA	1570	-1373 ~ +197
C	F3: **GG***GGTACC*CCACCTCGGAGTCCCTGTATTCT<br>R3: **CCC***AAGCTT*CCTTCAGGGAGAACGGAGCAGA	1101	-904 ~ +197
D	F4: **GG***GGTACC*GGAGCCCGCCCACGCAAGAT<br>R4: **CCC***AAGCTT*CCTTCAGGGAGAACGGAGCAGA	887	-690 ~ +197
E	F5: **GG***GGTACC*CTTCACCTTTCCACCCTCCCTC<br>R5: **CCC***AAGCTT*TGATGGCTGGTCCGGAATG	1721	-1799 ~ -79

Note: The transcription start site is indicated as +1; upstream is denoted with a negative number, and downstream is denoted with a positive number. The *Kpn*I and *Hin*dIII restriction sites are indicated in italics, and the attached nucleotides are indicated in bold.

Table S3 Prediction of the *TGFβ1* promoter

Programs	Promoter position	Comments
PROSCAN	-731 to -981<br>29 to 279	Promoter Score: 77.02 (promoter cutoff: 53.00)<br>Promoter Score: 78.22 (promoter cutoff: 53.00)
MatInspector	-447 to 340	Promoter Score: 0.714(promoter cutoff: 0.50)
Promoter 2.0	-1000<br>-430	Promoter Score: 0.571 (promoter cutoff: 0.50)
